# Clinical dementia severity associated with ventricular size is differentially moderated by cognitive reserve in men and women

**DOI:** 10.1186/s13195-018-0419-2

**Published:** 2018-09-05

**Authors:** Shraddha Sapkota, Joel Ramirez, Donald T. Stuss, Mario Masellis, Sandra E. Black

**Affiliations:** 1 Hurvitz Brain Sciences Research Program, Sunnybrook Research Institute, Sunnybrook Health Sciences Centre, 2075 Bayview Avenue, M6-192, Toronto, ON M4N 3M5 Canada; 2 Departments of Medicine, University of Toronto, 190 Elizabeth Street, R. Fraser Elliot Building, 3-805, Toronto, ON M5G 2C4 Canada; 3 Department of Psychology, University of Toronto, 100 St. George Street, 4th Floor, Sidney Smith Hall, Toronto, ON M5S 3G3 Canada; 4 Rotman Research Institute of Baycrest Centre, 3560 Bathurst Street, Toronto, ON M6H 4A6 Canada; 5 Department of Medicine (Neurology), University of Toronto, 190 Elizabeth Street, R. Fraser Elliot Building, 3-805, Toronto, ON M5G 2C4 Canada

**Keywords:** Cognitive reserve, Ventricular size, Sex, Cognitive impairment, Dementia, Sunnybrook Dementia Study

## Abstract

**Background:**

Interindividual differences in cognitive reserve (CR) are associated with complex and dynamic clinical phenotypes observed in cognitive impairment and dementia. We tested whether (1) CR early in life (E-CR; measured by education and IQ), (2) CR later in life (L-CR; measured by occupation), and (3) CR panel (CR-P) with the additive effects of E-CR and L-CR, act as moderating factors between baseline ventricular size and clinical dementia severity at baseline and across 2 years. We further examined whether this moderation is differentially represented by sex.

**Methods:**

We examined a longitudinal model using patients (*N* = 723; mean age = 70.8 ± 9.4 years; age range = 38–90 years; females = 374) from the Sunnybrook Dementia Study. The patients represented Alzheimer’s disease (*n* = 439), mild cognitive impairment (*n* = 77), vascular cognitive impairment (*n* = 52), Lewy body disease (*n* = 30), and frontotemporal dementia (*n* = 125). Statistical analyses included (1) latent growth modeling to determine how clinical dementia severity changes over 2 years (measured by performance on the Dementia Rating Scale), (2) confirmatory factor analysis to establish a baseline E-CR factor, and (3) path analysis to predict dementia severity. Baseline age (continuous) and *Apolipoprotein E* status (ɛ4−/ɛ4+) were included as covariates.

**Results:**

The association between higher baseline ventricular size and dementia severity was moderated by (1) E-CR and L-CR and (2) CR-P. This association was differentially represented in men and women. Specifically, men in only the low CR-P had higher baseline clinical dementia severity with larger baseline ventricular size. However, women in the low CR-P showed the (1) highest baseline dementia severity and (2) fastest 2-year decline with larger baseline ventricular size.

**Conclusions:**

Clinical dementia severity associated with ventricular size may be (1) selectively moderated by complex and additive CR networks and (2) differentially represented by sex.

**Trials registration:**

ClinicalTrials.gov, NCT01800214. Registered on 27 February 2013.

**Electronic supplementary material:**

The online version of this article (10.1186/s13195-018-0419-2) contains supplementary material, which is available to authorized users.

## Background

The spectrum of neurodegenerative pathologies in the aging brain in combination with cardiovascular risk factors has led to an increased prevalence and diagnosis of mixed neurodegenerative diseases (combination of proteinopathies and vasculopathies) [1]. A recent review examined 12 community-based neuropathology studies and identified that the frequency of Alzheimer’s disease (AD) pathology was 19–67%, Lewy body pathology was 6–39%, vascular pathologies was 28–70%, and mixed pathologies was 10–74% [2]. Combinations of neurodegenerative (e.g., proteinopathies) and non-neurodegenerative (e.g., metabolic-nutritional, vascular) pathologies may depict the multifaceted clinical manifestations observed in patients with dementia. In addition to the heterogeneity and complexity of pathologies, variability and differences in life experiences may further complicate the course of cognitive decline and clinical dementia severity. Identifying modifiable risk and protective proxies of cognitive reserve (CR) may lead to personalized interventions for those most at risk of accelerated cognitive impairment [3]. Cognitive resilience is another related and complementary hypothetical construct and term used in the literature [4].

CR is defined as the ability to compensate for neurological damage via recruitment of compensatory mechanisms [5, 6]. Diverse life experiences, including level of education [7], occupational attainment [8], involvement in leisure activities [9], and IQ, are commonly used as proxies to indirectly represent CR [10]. Many of these variables are intercorrelated; for example, higher IQ leads to more education [11]. However, independent associations have also been observed between each variable and cognitive decline [12]. Inconsistent findings between CR proxies and cognition imply a potentially elaborate CR network. Such intricate CR networks and pathology in the aging brain may be further convoluted by sex differences in AD [13], Parkinson’s disease [14], vascular dementia [15], and frontotemporal dementia (FTD) [16]. For example, men diagnosed with AD show better performance on language, semantic and visuospatial tasks, and episodic memory than women after accounting for age, education, and dementia severity [17]. Estrogen deficiency in postmenopausal women may partly explain this disadvantage observed in women [18]. On the contrary, women may have an advantage in verbal memory even in the presence of hippocampal atrophy [19, 20].

In the present study, we examine whether the association between ventricular size and clinical dementia severity [21–23] is differentially moderated by CR in men and women across a large, heterogeneous sample of patients with cognitive impairment and dementia. We differentiate CR into two constructs: (1) CR acquired early in life (E-CR) measured with education and IQ as proxies and (2) CR acquired through maintenance or practice later in life (L-CR) measured with occupational attainment as a proxy, likely capturing what is referred to by some as “maintenance” [24, 25]. Our approach examines whether there are differences in CR moderation for (1) early life, (2) later life, and (3) the additive effects of E-CR + L-CR with the CR panel (CR-P). The synergistic associations of high E-CR and high L-CR may provide additional protection against neuropathological damage in cognitive impairment and dementia.

The rationale for differentiating the two constructs derive from studies focusing on occupation, education, and IQ separately as proxies for CR. Occupation has independently been linked to cognitive performance in multiple domains (memory, processing speed, attention) after accounting for potential confounding factors such as education, leisure activities, and sex differences [26]. Authors of a recent systematic review observed inconsistencies between occupation and general cognition in the presence of multiple brain pathologies in different clinical groups [27]. In men, higher intellectual and more engaging work was associated with better cognitive performance in late life independent of IQ or education [28]. Occupation, often characterized as nonformal education, is training in a particular skill set that can develop and evolve across the lifespan [29]. This suggests that the dynamic fluctuations and continually evolving proxy for CR across the lifespan is better represented by occupation, whereas IQ and education may be more accurate proxies for CR typically acquired in early life. We therefore examined (1) occupation as an independent factor contributing to the CR network to account for any changes, development, and skills acquired across the lifespan, including later in life (L-CR), and (2) education and IQ for CR acquired early in life that may not have been actively exercised or preserved in a stimulating work environment throughout the life course (E-CR).

In addition to commonly used CR proxies, CR has also been studied in the context of both functional and structural imaging. Functional imaging studies have commonly been used to capture CR by examining differential activation patterns [29, 30]. AD patients with high CR (indexed by education) may have higher functional connectivity in the posterior cingulate cortex. Specifically, higher activation of compensatory neural mechanisms in this region may mitigate the effects of greater AD pathology [31]. Structural imaging research has also shown that (1) lower socioprofessional attainment in midlife was associated with faster hippocampal atrophy [32], (2) interaction between white matter lesions and high CR predicted higher conversion rate of mild cognitive impairment (MCI) to AD [33], and (3) larger intracranial volume provided support for brain reserve hypothesis against AD pathology [34]. CR may account for discrepancies observed between sex, lifestyle factors, AD neuropathological changes, small-vessel vascular impairment, and dementia with Lewy bodies (DLB) [35].

Brain pathologies commonly represented by neuroimaging biomarkers (e.g., ventricular atrophy, white matter hyperintensities) provide a baseline to predict cognitive decline [36, 37] and risk of dementia [38]. We specifically focus on ventricular volume in the present study because previous research has shown that (1) ventricular volume is an important marker in predicting conversion from MCI to AD [39], (2) ventricular expansion is accelerated 24 months prior to signs of cognitive deficits in MCI participants [40], and (3) larger ventricular volumes may indicate higher risk of clinical dementia severity or related pathology [41]. However, the impact of a CR network and sex differences should be studied in conjunction with ventricular volume to understand the neural underpinnings associated with rate of cognitive deterioration in dementia. The present study includes a unique cohort of cognitive impairment and dementia patients recruited from a real-world tertiary clinical setting. The patients represent a wide clinical spectrum of typical neuropathology, including AD, MCI, vascular cognitive impairment (VCI), FTD, DLB, and combined pathologies (e.g., AD + VCI, AD + DLB, AD + VCI).

### Research questions

**Fig. 1:**
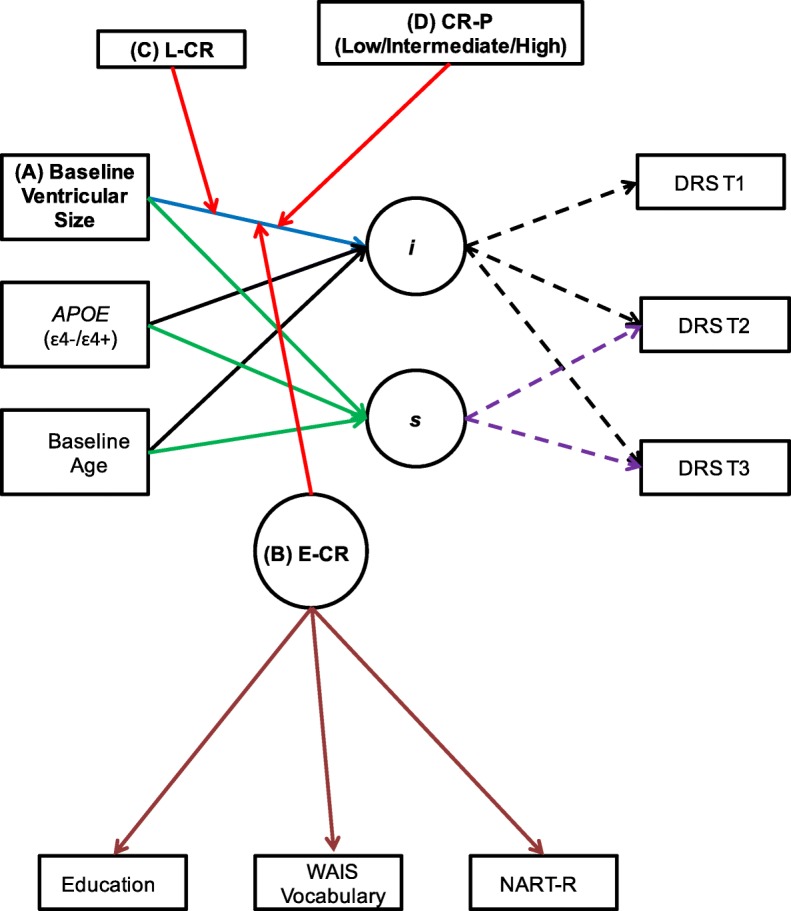
Conceptual model for baseline ventricular size on baseline Dementia Rating Scale (DRS) performance and 2-year change (A) independently, (B) as moderated by early-life cognitive reserve (E-CR), (C) as moderated by later-life cognitive reserve (L-CR), and (D) as moderated by cognitive reserve panel (CR-P = E-CR + L-CR). *Apolipoprotein E *(*APOE*) genotype and baseline age are included as covariates in all analyses. *i* Intercept, *s* Slope, *T* Time point, *WAIS* Wechsler Adult Intelligence Scale, *NART* North American Reading Test-Revised

We examined four main research questions. Overall, we tested whether ventricular size [40] (an overall global measure of atrophy [42, 43]) predicts clinical dementia severity using the Dementia Rating Scale (DRS) (a commonly used scale to stage clinical dementia severity [44, 45]) at baseline and over 2 years. A longitudinal design was used for baseline DRS scores and two follow-up visits at 12 months and 24 months. Any associations were further tested for CR moderation and stratified by sex (see Fig. 1).

#### Research question 1 (RQ1)

Does higher baseline ventricular size predict poorer baseline DRS performance and steeper 2-year decline? On the basis of previous research, we expected to first establish this association in our present dementia sample.

#### Research question 2 (RQ2)

Does E-CR moderate clinical dementia severity for ventricular size such that those with higher ventricular size and lower E-CR have poorer DRS performance and steeper decline than those with a higher level of E-CR? We also examined whether this association is magnified in women compared with men.

#### Research question 3 (RQ3)

Does L-CR moderate clinical dementia severity for ventricular size such that those with higher ventricular size and lower L-CR have poorer DRS performance and steeper decline than those with higher levels of L-CR? We also examined whether this moderation is magnified in women compared with men.

#### Research question 4 (RQ4)

Does CR-P moderate clinical dementia severity for ventricular size such that those with higher ventricular size in the low CR-P group have poorer DRS performance and steeper 2-year decline than those in the high CR-P group? We also examined whether this moderation is differentially represented by sex. We expected that any moderating effects between higher baseline ventricular size and higher dementia severity and accelerated increase (steeper 2-year DRS decline) would be magnified in the low CR-P panel, particularly in women.

## Methods

### Participants

We used data from the Sunnybrook Dementia Study (SDS) [46], a large, longitudinal, observational, prospective cohort study (1994–present) of patients with dementia in Toronto, Canada, that includes clinical, standardized neuroimaging, neuropsychological, functional, mood, behavioral, and genetic assessments (ClinicalTrials.gov, NCT01800214). Patients and control subjects (*N* = 1498) were recruited from the Cognitive Neurology Clinic at Sunnybrook Health Sciences Centre, University of Toronto, Canada. All patients were enrolled through physician referrals and cognitively normal control individuals through word of mouth or advertisements. The recruited patients represented AD [47, 48], MCI [49, 50], VCI [51], DLB [52], FTD [53, 54], and mixed neurodegenerative diseases. Human/institutional research ethics guidelines were met in full for all SDS and ongoing data collection procedures. Written informed consent was obtained from all participants. For the present sample, we applied the following exclusionary criteria: (1) all cognitively normal control individuals, (2) participants who did not have data on the DRS, and (3) participants who did not have baseline ventricular size and total intracranial volume data. Accordingly, a total of 723 patients (age range = 38–90 years; mean age = 70.77 [9.42] years; females = 374) were included in this analysis. The patients represented were AD (*n* = 439), MCI (*n* = 77), VCI (*n* = 52), DLB (*n* = 30), and FTD (*n* = 125) (Table 1). Any mixed neurodegenerative cases were grouped into the dominant clinical group.

**Table 1:** Baseline participant characteristics by clinical status

Characteristics	AD	MCI	VCI	FTD	DLB	Total
*n*	439	77	52	125	30	723
Age, years	71.7 (9.3)	70.3 (8.2)	71.1 (8.2)	66.9 (9.1)	73.2 (8.8)	70.8 (9.4)
Sex, F/M	233/206	47/30	20/32	64/61	10/20	374/349
MMSE	23.0 (4.8)	27.6 (1.9)	25.7 (3.9)	23.1 (6.0)	23.9 (4.4)	23.8 (4.9)
Ventricular size^a^, cm^3^	43.4 (19.9)	31.4 (17.2)	45.6 (24.5)	42.5 (21.2)	42.7 (20.0)	42.1 (20.5)
WMH^a^, cm^3^	7.8 (11.1)	3.3 (4.8)	13.2 (17.5)	4.6 (6.8)	7.2 (11.6)	7.1 (10.8)
TIV, cm^3^	1213.6 (140.2)	1198.0 (121.8)	1225.1 (122.3)	1204.9 (121.1)	1269.5 (127.3)	1213.6 (133.7)
Dementia Rating Scale	117.8 (14.8)	133.5 (7.8)	126.4 (13.7)	115.7 (19.3)	118.7 (14.6)	119.9 (15.9)
NART-R	107.4 (9.9)	110.1 (9.1)	108.3 (10.3)	102.3 (11.0)	110.0 (10.4)	107.1 (10.3)
WAIS vocabulary	42.8 (14.1)	51.3 (9.8)	42.2 (12.5)	34.7 (18.2)	44.8 (13.3)	42.7 (14.5)
Education, years	13.8 (3.8)	14.0 (3.4)	13.7 (3.9)	14.1 (3.9)	13.9 (3.4)	13.9 (3.7)

*Abbreviations: AD* Alzheimer’s disease, *DLB* Dementia with Lewy bodies, *FTD* Frontotemporal dementia, *MCI* Mild cognitive impairment, *MMSE* Mini Mental State Examination, *NART-R* National Adult Reading Test–Revised, *TIV* Total intracranial volume, *VCI* Vascular cognitive impairment, *WAIS* Wechsler Adult Intelligence Scale, *WMH* White matter hyperintensities

^a^Corrected for total intracranial volume

### Magnetic resonance imaging acquisition protocols and processing

Structural brain images were obtained using a 1.5-T Signa system (GE Healthcare Life Sciences, Milwaukee, WI, USA). Ventricular size was calculated as the ventricular cerebrospinal fluid compartment using the Lesion Explorer (LE) pipeline and Semi-Automatic Brain Region Extraction (SABRE) tools [55, 56]. Complete details on the LE pipeline and SABRE are available elsewhere [55, 57]. For the present study, all baseline ventricular size measurements were corrected with total intracranial volume (ICV) for each participant (baseline ventricular size/baseline ICV).

### Cognitive reserve

We examined two standard and widely used measures of intelligence and years of education as proxies for E-CR factor [6, 10, 58, 59] and occupation [60] as proxy for L-CR.

#### Wechsler Adult Intelligence Scale vocabulary

This verbal IQ test [61] consists of a list of 35 words. Participants are asked the meaning of each word starting at item 4. Each item receives a score of 0 (incorrect meaning), 1 (a vague answer), or 2 (correct meaning). Participants receive 2 points each for items 1–3 if items 4–8 received a score of 1 or 2. The test is discontinued after five consecutive failures. The final score is out of 70, with higher scores representing better performance.

#### North American Adult Reading Test–Revised

This verbal IQ test [62] consists of 61 words in four columns. Participants are asked to read each word aloud, going down the list for the two columns on the first page and continuing on to the next page. The number of errors is recorded. The final scaled score is computed with the formula = 127.8–0.78 (number of errors), with higher scores representing better performance.

#### Education

Years of total education [63] completed was recorded and used as a continuous scale. Years of education ranged from 0 to 32. Higher number of years represented higher levels of education.

#### Hollingshead Index of Social Position

Participants were asked their current occupation. For the present study, we examined only the occupation scale [60], which has a total weight of 7, with lower scores representing higher executive and major professionals and higher scores for those with hard physical work/labor (*see* Additional file 1: Table S1).

### Clinical dementia severity

#### Mattis Dementia Rating Scale

This test [64] is widely used to measure clinical dementia severity in older adults and dementia patients [44, 65, 66]. The test consists of five subscales: (1) attention - 37 points; (2) initiation and preservation - 37 points, (3) construction - 6 points, (4) conceptualization - 39 points, and (5) memory - 25 points. Participants are asked to correctly perform different tasks within each subscale. The final score is from 0 to 144, with lower scores reflecting poorer performance (i.e., higher dementia severity).

### Statistical analyses

Structural equation modeling was used for all analyses with Mplus version 7.4 [67]. All missing values for E-CR measures were assumed to be missing at random and were estimated using maximum likelihood. Cases with missing predictor values were removed using list-wise deletion in Mplus 7.4.

First, we used confirmatory factor analysis to examine loadings of three manifest variables (Wechsler Adult Intelligence Scale [WAIS] vocabulary, North American Adult Reading Test–Revised [NART-R], and education) on the predicted one-factor E-CR latent variable. All three observed variables were tested to determine the best fit and parsimonious model. The best fit model was determined by examining several fit statistics. The chi-square test of model (χ^2^; *p* > 0.05) allowed for an overall indication of good model fit. Additional absolute/comparative fit indices were also examined to determine a good model fit to the data [68]: root mean square error of approximation (RMSEA ≤ 0.05), comparative fit index (CFI ≥ 0.95), and standardized root mean square residual (SRMR ≤ 0.08).

Second, we used latent growth modeling to determine how DRS performance changes over 2 years. We adopted a model-building approach, started with a simple (null) model, and added parameters at each step to arrive at a baseline model of change. The null model assumes that there is no change over the three time points, followed by the addition of fixed intercepts, random intercepts, fixed slope, random slope, and fixed quadratic. In the null model, the variances for the intercepts were fixed across participants to 0. In the random intercepts model, individuals were allowed to vary in intercept variance by removing the fixed intercept at 0. A fixed linear slope was added to the baseline model by fixing the slope to 0 across all participants. The fixed linear slope assumed that all participants were changing in performance at the same rate. Participants were allowed to vary in their slope performance by removing the fixed linear slope constraint and adding a random intercept and random linear slope model of change. A fixed quadratic was added to the random intercept and random linear slope models, where both the intercepts and the slope were allowed to vary across individuals, but the curvilinear change was fixed across all participants. Following the examination of model fit, the *χ*^2^difference statistic was calculated to detect any improvement in fit with the addition of free parameters at each step.

Third, to examine moderating effects, we created interaction terms between E-CR and ventricular size to examine E-CR moderation (RQ2) and L-CR and ventricular size to examine L-CR moderation (RQ3). We further examined both interactions stratified by sex. To test CR-P moderation and account for any sex differences, we dichotomized E-CR and L-CR into low and high and summed accordingly for the CR-P groups. This stratification approach to test moderation in Mplus is common and widely accepted (see [69–72]). Specifically, we used the same cutoff for low and high E-CR and L-CR groups in both sexes. We dichotomized (1) E-CR factor score into low versus high by splitting at the overall mean factor score, (2) L-CR with a median split (3.5) of occupational status into high (less than 3.5) versus low (greater than 3.5) groups, and (3) CR-P by subtracting across all low versus high dichotomized groups with E-CR factor (0 = low; 1 = high) – L-CR (0 = high; 1 = low). All possible combination scores of − 1 (low E-CR + low L-CRCR), 0 (low E-CR + high L-CR or high E-CR + low L-CR), and 1 (high E-CR + high L-CR) were represented as low, intermediate, and high CR-P groups, respectively.

Fourth, the best DRS latent growth model was regressed on baseline ventricular size (RQ1) independently, (RQ2) with E-CR × ventricular size, E-CR, ventricular size and as stratified by sex, (RQ3) with L-CR × ventricular size, L-CR, ventricular size and as stratified by sex, and (RQ4) moderated by CR-P (low versus intermediate versus high) and as stratified by sex. Baseline age (continuous) and *APOE* (ɛ4−/ɛ4+) status [73–77] were included as covariates in all analyses. One hundred ninety-eight participants with missing values on apolipoprotein E (*APOE*) genotype and an additional fifteen participants with missing values on all three DRS time points were lost owing to list-wise deletion. For model fit statistics and significant results, we examined the regression estimate and *p* < 0.05.

## Results

Descriptive characteristics for all participants by dementia classification are reported in Table 1. One-factor E-CR factor with WAIS vocabulary, NART-R, and education provided the best model fit [*χ*^2^(*df*) = 1.121 (1), *p* = 0.299; RMSEA (90% CI) = 0.013 (0.000–0.101); CFI = 1.000; SRMR = 0.062]. We computed factor scores for the E-CR latent factor, which was used in all succeeding analyses to examine moderation. For the DRS latent growth model, the random intercept and random slope model provided the best model fit (*see* Table 2) and was used in all subsequent analyses.

**Table 2:** Latent growth model fit statistics and chi-square difference test for Dementia Rating Scale

Model	${\chi}_M^2$(*df*_*M*_)	CFI	RMSEA (90% CI)	SRMR	${\chi}_D^2$ (*df*_*D*_)
Fixed intercept	587.826(5)**	0.000	0.409 (0.381–0.437)	0.801	–
Random intercept	264.295(4)**	0.535	0.306 (0.275–0.337)	0.577	323.531(1)**
Random intercept, fixed slope	76.559(3)**	0.869	0.188 (0.153–0.225)	0.402	187.736(1)**
Random intercept, random slope	0.967(1)	1.000	0.000 (0.000–0.099)	0.033	75.592(2)**
Random intercept, random slope, fixed quadratic	0.000(0)**	1.000	0.000 (0.000–0.000)	0.000	0.967(1)
Random intercept, random slope, random quadratic	No convergence				

${\chi}_M^2$ Chi-square test of model fit, *df*_*M*_ Degrees of freedom for model fit, *RMSEA* Root mean square error of approximation, *CFI* Comparative fix index, *SRMR* Standardized root mean square residual, ${\chi}_D^2$ Chi-square test of difference, *df*_*D*_ Degrees of freedom for chi-square difference test

**p < 0.001

For RQ1, we observed that larger baseline ventricular size was associated with poorer DRS performance (β = − 0.202, SE = 0.033, *p* < 0.001) and predicted steeper 2-year decline (β = − 0.085, SE = 0.026, *p* = 0.001) in the overall group.

For RQ2, we observed two significant effects of E-CR moderation between baseline ventricular size and DRS performance. First, the negative impact of lower E-CR on DRS performance was more pronounced in patients with larger ventricular size (β = − 0.093, SE = 0.032, *p* = 0.004). Second, when stratified by sex, this significant association was present only in men. Specifically, the negative impact of lower E-CR on DRS performance was present only in men with larger baseline ventricular size (β = − 0.104, SE = 0.041, *p* = 0.012). We did not observe that E-CR moderated the association between baseline ventricular size and DRS performance or 2-year change in women.

For RQ3, we observed two significant effects of L-CR moderation. First, the negative impact of lower L-CR on DRS performance was more pronounced in patients with larger baseline ventricular size (β = − 0.052, SE = 0.021, *p* = 0.014). Second, when stratified by sex, this significant association was present only in women. Specifically, the negative impact of lower L-CR on DRS performance was present only in women with larger baseline ventricular size (β = − 0.086, SE = 0.034, *p* = 0.012). We did not observe that L-CR moderated the association between baseline ventricular size and DRS performance or 2-year change in men.

**Fig. 2:**
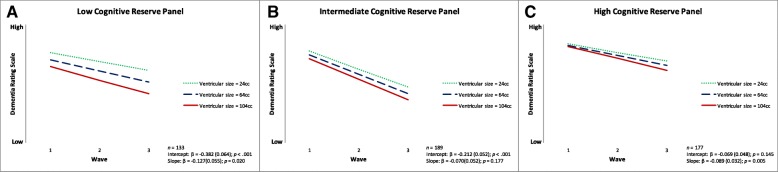
Predicted growth curves for baseline ventricular size on Dementia Rating Scale performance and 2-year change as moderated by cognitive reserve panel (early-life cognitive reserve + later-life cognitive reserve) in the overall group: (**a**) low, (**b**) intermediate, and (**c**) high

**Fig. 3:**
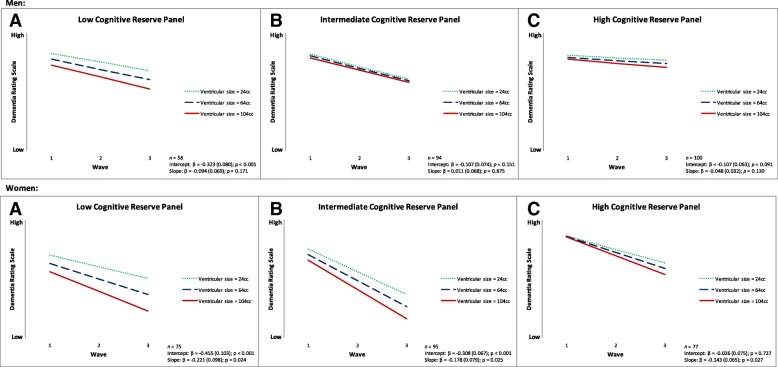
Predicted growth curves for baseline ventricular size on Dementia Rating Scale performance and 2-year change as moderated by cognitive reserve panel (early-life cognitive reserve + later-life cognitive reserve) in men and women: (**a**) low, (**b**) intermediate, and (**c**) high

For RQ4, we observed three significant associations. First, CR-P moderated the association between baseline ventricular size and DRS performance and change. Specifically, larger baseline ventricular size was associated with poorer baseline DRS performance in the low (β = − 0.382, SE = 0.064, *p* < 0.001) and intermediate (β = − 0.212, SE = 0.052, *p* < 0.001) CR-P groups and steeper 2-year decline in the low (β = − 0.127, SE = 0.055, *p* = 0.020) and high (β = − 0.089, SE = 0.032, *p* = 0.005) CR-P group overall (Fig. 2). Second, when stratified by sex, larger baseline ventricular size in men was associated with poorer performance (β = − 0.323, SE = 0.080, *p* < 0.001) only in the low CR-P group. Men in all three groups did not show 2-year DRS decline with larger baseline ventricular size. Third, in women, larger baseline ventricular size was associated with poorer performance in the low (β = − 0.455, SE = 0.103, *p* < 0.001) and intermediate (β = − 0.308, SE = 0.067, *p* < 0.001) CR-P groups (*see* Table 3; Fig. 3). For 2-year decline, women with larger baseline ventricular size showed steeper decline in the low (β = − 0.221, SE = 0.098, *p* = 0.024), intermediate (β = − 0.178, SE = 0.079, *p* = 0.025), and high (β = − 0.143, SE = 0.065, *p* = 0.027) CR-P groups (*see* Table 3; Fig. 3).

**Table 3:** Unstandardized regression coefficients and model fit indices for all models examining baseline ventricular size on Dementia Rating Scale performance and 2-year change

Models	Intercept			Linear slope			Model fit indices			
	β	SE	*p* Value	β	SE	*p* Value	${\upchi}_{\mathrm{M}}^2$(df_M_)	CFI	RMSEA (90% CI)	SRMR
Independent (*n* = 510)	− 0.202	0.033	0.000	− 0.085	0.026	0.001	13.258 (6), *p* = 0.039	0.986	0.049 (0.010–0.085)	0.034
E-CR moderation (*n* = 510)	− 0.093	0.032	0.004	− 0.049	0.028	0.079	5.582 (7), *p* = 0.589	1.000	0.000 (0.000–0.047)	0.013
E-CR moderation by sex										
Male (*n* = 254)	− 0.104	0.041	0.012	− 0.046	0.033	0.167	6.737 (7), *p* = 0.457	1.000	0.000 (0.000–0.075)	0.035
Female (*n* = 256)	− 0.076	0.054	0.159	− 0.046	0.052	0.378	5.499 (8), *p* = 0.703	1.000	0.000 (0.000–0.056)	0.045
L-CR moderation (*n* = 499)	− 0.052	0.021	0.014	0.016	0.015	0.296	7.308 (7), *p* = 0.398	0.999	0.009 (0.000–0.056)	0.012
L-CR moderation by sex										
Male (*n* = 252)	− 0.033	0.027	0.218	0.015	0.017	0.359	7.265 (7), *p* = 0.402	0.999	0.012 (0.000–0.079)	0.034
Female (*n* = 247)	− 0.086	0.034	0.012	0.010	0.033	0.764	7.200 (8), *p* = 0.515	1.000	0.000 (0.000–0.070)	0.024
CR-P moderation							31.751 (18), *p* = 0.024	0.973	0.068 (0.025–0.106)	0.058
(a) Low (*n* = 133)	− 0.382	0.064	0.000	− 0.127	0.055	0.020				
(b) Intermediate (*n* = 189)	− 0.212	0.052	0.000	− 0.070	0.052	0.177				
(c) High (*n* = 177)	− 0.069	0.048	0.145	− 0.089	0.032	0.005				
CR-P moderation by sex										
Male							32.143 (18), *p* = 0.021	0.941	0.097 (0.037–0.150)	0.141
(a) Low (*n* = 58)	− 0.323	0.080	0.000	− 0.094	0.069	0.171				
(b) Intermediate (*n* = 94)	− 0.107	0.074	0.151	0.011	0.068	0.875				
(c) High (*n* = 100)	− 0.107	0.063	0.091	− 0.048	0.032	0.130				
Female							12.656 (18), *p* = 0.817	1.000	0.000 (0.000–0.062)	0.078
(a) Low (*n* = 75)	− 0.455	0.103	0.000	− 0.221	0.098	0.024				
(b) Intermediate (*n* = 95)	− 0.308	0.067	0.000	− 0.178	0.079	0.025				
(c) High (*n* = 77)	− 0.026	0.075	0.727	− 0.143	0.065	0.027				

*Abbreviations: β* Regression estimate, ${\chi}_M^2$ Chi-square test of model fit, *df*_*M*_ Degrees of freedom for model fit, *CFI* Comparative fix index, *CR-P* Cognitive reserve panel (E-CR + L-CR), *E-CR* Early-life cognitive reserve, *L-CR* Later-life cognitive reserve, *RMSEA* Root mean square error of approximation, *SRMR* Standardized root mean square residual

## Discussion

We examined whether the association between baseline ventricular size and clinical dementia severity (measured with DRS) is differentially moderated by CR in men and women. This is the first study to show that (1) CR-P (an additive panel of E-CR and L-CR) (2) moderates the association between baseline ventricular size and DRS performance and 2-year change (3) in a heterogeneous dementia population (AD, MCI, VCI, DLB, FTD, mixed pathologies). We observed two main findings. First, we established a baseline association between ventricular size and dementia severity in our mixed dementia sample. Specifically, higher baseline ventricular size was associated with poorer baseline DRS performance and steeper 2-year decline. Second, we observed that this association is moderated by (1) E-CR and L-CR (occupational attainment) in the overall group and differentially in men and women and (2) CR-P in the overall group and differentially in men and women. Specifically, women in all three CR-P groups had steeper 2-year DRS decline with larger baseline ventricular size, whereas men in the same groups were not declining at a significant rate.

Our novel approach and findings suggest that (1) it is imperative to account for both the independent and additive influences of E-CR and L-CR and sex differences in conjunction with clinical symptoms and neuroimaging biomarkers to understand the underlying neuropathology of clinical phenotypes in dementia and (2) understanding this complex synergistic association may lead to earlier detection and personalized interventions by identifying subgroups at high risk. Specific comments related to our findings by RQs follow.

For RQ1, the main result was that larger baseline ventricular size was associated with poorer DRS performance and steeper 2-year decline. As expected and observed in previous studies [78], this study establishes the link between subcortical atrophy (i.e., ventricular size [22]) and cognitive impairment in sampling across different dementias in the present sample. We observed that baseline ventricular size alone is an important predictor of cognitive impairment and dementia severity. Supporting recent findings with other brain structures include (1) hippocampal atrophy was associated with lower scores on the Montreal Cognitive Assessment [79], (2) white matter hyperintensities were associated with performance on the Boston Naming Test and the Trail Making Test B [80], (3) ventricular size and hippocampal volume predicted dementia incidence [81], and (4) baseline volume of the right hippocampus and entorhinal cortex were associated with time to preclinical AD incidence [82]. Our findings extend previous literature on brain atrophy and cognition in normal aging older adults and pure dementia cases to a unique sample representing a range of dementias.

For RQ2, we observed that E-CR latent factor moderated the association between baseline ventricular size and baseline dementia severity in the overall group. However, when stratified by sex, the negative effect of lower E-CR on DRS performance was present only in men with larger baseline ventricular size. This suggests that CR typically obtained in early life (IQ and education) alone may not be an important independent moderator between ventricular size and dementia severity in both sexes. Because this is the first study to examine for interactive associations between E-CR factor and ventricular size sampling from different dementia groups, our goal was to test a general cognitive functioning domain. In this regard, we chose the DRS, which included subscales for memory, attention, preservation, construction, and conceptualization [64]. Future studies should consider examining moderation effects of E-CR between other neuroimaging biomarkers (e.g., hippocampal atrophy, white matter hyperintensities) and specific cognitive domains (e.g., memory, neurocognitive speed) in pure cognitive impairment and dementia cases (e.g., MCI or AD [80, 83]).

For RQ3, L-CR moderated the association between baseline ventricular size and DRS performance. Specifically, the negative effect of lower L-CR on DRS performance was more pronounced in patients with larger baseline ventricular size. When stratified by sex, this association was only present in women. This sex difference for L-CR suggests that women with lower occupational status may be more vulnerable to higher dementia severity than men. We examined occupation as a measure of L-CR that is obtained across the lifespan because previous work has shown that higher levels of occupational complexity among adults at risk for dementia and with similar levels of cognition were associated with lower hippocampal volume and higher overall brain atrophy [84]. Lower socioprofessional attainment in midlife and across the life course has also been linked to faster rate of hippocampal atrophy [32]. In the present study, this implies that adults with higher occupational status and complexity may be using CR accumulated across the lifespan. This interesting and novel finding implies that L-CR with occupation as a proxy may be more important for women in the context of larger baseline ventricular size. Previous work examining neuropathological changes in men and women has shown similar levels of AD- and FTD-related dementia severity, despite men having significantly reduced cerebral metabolic glucose rate (an indication of greater CR) [16, 85]. Future studies may benefit from examining sex differences when studying the influence of neuroimaging biomarkers on dementia severity as moderated by dynamic CR networks.

For RQ4, CR-P moderated the association between baseline ventricular size and DRS performance and 2-year decline. Specifically, larger baseline ventricular size was associated with poorer baseline DRS performance in the low and intermediate CR-P groups, as well as with steeper 2-year DRS decline in the low and high CR-P groups. Although we expected to observe significant decline only in the low L-CR group, our significant finding is consistent with the literature showing that older adults with higher CR show higher resilience and plasticity to cognitive impairment [86]. Patients with dementia with high CR factors are more likely to have greater brain pathology and vulnerability triggering a rapid deterioration in cognition once a critical threshold is reached [87]. This threshold effect may be more common in clinically diagnosed cases with the presence of additional neuronal pathologies than in cognitively normal older adults [80, 88]. Our finding demonstrates that the additive effects of E-CR and L-CR may differentially contribute to the complex clinical manifestations observed in dementia. Future studies should consider testing the relationship between E-CR and L-CR in patients with dementia to target intervention programs at a younger age.

Previous studies have observed that cognitively normal older adults with high IQ and high socioeconomic status show less cognitive impairment in the presence of hippocampal atrophy [79], and early education and intellectual activities may protect against amyloid beta deposition in late life [89]. Consistent with these other studies, we observed that dementia patients in the high CR-P group were protected from poorer baseline DRS performance with larger baseline ventricular size but showed steeper 2-year DRS decline. The protective effects of high CR-P were present at baseline, but this association was absent for change over time on the DRS. Two forms of underlying neural networks are commonly linked to CR: (1) neural reserve, reserve associated with preexisting cognitive networks, and (2) neural compensation, the ability to recruit compensatory mechanisms [87]. Findings on neural networks have observed that older adults with high CR may have reduced neural reserve and increased neural compensation compared with younger adults with high CR [87]. Our present findings extend this literature by (1) showing that clinically diagnosed older adults with high CR-P maybe recruiting more neural mechanisms at baseline to compensate for the higher baseline subcortical atrophy, whereas those with low CR-P are not able to perform at the same level, and (2) this protective association of high CR-P despite larger baseline ventricular size on DRS performance dissipates across 2 years, resulting in greater dementia severity.

We also observed differential interactions between the three CR-P groups and baseline ventricular size on DRS performance in men and women. Both men and women in the low CR-P groups showed greater dementia severity. However, only women in the intermediate group had greater dementia severity with larger baseline ventricular size, but men in the same group did not show this association. Regarding steeper 2-year decline, men did not show decline on the DRS with larger baseline ventricular size in any of the of three (low, intermediate, high) CR-P groups. However, women in all three CR-P groups had steeper 2-year DRS decline with larger baseline ventricular size. A major biological difference between older men and women is reduced estrogen levels in postmenopausal women, which may lead to increased dementia risk [18]. Estrogen, primarily produced by the corpus luteum and the placenta, influences receptors in the hippocampus, basal forebrain, and cerebral cortex. Estrogen has also been shown to be important for growth and survival of cholinergic neurons, antioxidant properties, and increased metabolism of amyloid precursor protein [18]. Future studies may benefit from studying the relationship between estrogen replacement therapy [17] and CR networks in older women at high risk of dementia to identify potential underlying mechanisms.

Furthermore, we observed that the longitudinal association between larger baseline ventricular size and steeper 2-year DRS decline in women was magnified in the low CR-P group (β = − 0.221) compared with the high group (β = − 0.143) (Table 3; Additional file 1: Table S2). This implies a faster rate of decline for women with low CR-P than for women with high CR-P. This novel finding emphasizes the need to aim for sex-specific intervention programs.

We note several strengths and limitations of the present study. First, we examined overall baseline ventricular size, whereas future studies may benefit from examining differences in specific brain regions contributing to ventricular size as well as longitudinal change in ventricular size [90]. Future studies should also consider other neuroimaging biomarkers targeting specific clinical diagnoses, such as hippocampal atrophy as a potential biomarker for AD [91]. Second, previous studies have shown that age and *APOE* ɛ4 status may play a role in dementia [81]. We included both as covariates in all our analyses and performed post hoc analyses to examine whether *APOE* genotype (ɛ4− versus ɛ4+) predicted baseline ventricular volume in our sample. *APOE* genotype did not influence baseline ventricular size (β = − 1.074, SE = 1.807, *p* = 0.552) in our sample (Additional file 1: Table S3). Future studies should consider looking at specific networks of genetic risks identified in recent genome-wide association studies and as stratified by *APOE* ɛ4 groups to investigate cognitive associations in both preclinical [71] and clinical cases. Third, traditionally, premorbid IQ is used as a proxy for CR [87], and we note this as a potential limitation in our study. IQ in the present study was measured at baseline when patients with mild dementia were recruited. Fourth, although we had a robust measure of occupational status (using the Hollingshead Index of Social Position), future studies should also include occupational complexity [84, 92] in addition to occupational status to examine L-CR and additive effects of other established proxies of reserve (e.g., lifestyle and physical activities, financial management [93]). Fifth, because we used the first three DRS measurements to examine change in dementia severity over a 2-year period, we acknowledge that additional time points may have resulted in a random quadratic or a different growth model [68]. Future studies should consider examining additional time points to address long-term changes in dementia severity.

There are also several strengths of the present study. First, our sample provides a unique representation of patients with dementia across a range of neurodegenerative diseases in a real-world tertiary clinic setting. We include this heterogeneous dementia population because the association of ventricular size with dementia severity may differ across all represented neurodegenerative conditions. As part of post hoc analyses, we excluded the MCI group to examine the association between ventricular size and dementia severity as moderated by CR-P in men and women. Similar to the overall group, this MCI-excluded group showed poorer baseline DRS performance with larger ventricular size in the low (*p* < 0.001) and intermediate (*p* = 0.001) CR-P groups and steeper 2-year decline in the low (*p* = 0.031) and high (*p* = 0.011) CR-P groups. For men, we observed poorer baseline DRS performance with larger ventricular size in only the low (*p* < 0.001) CR-P group. For women, we observed poorer baseline DRS performance with larger ventricular size in the low (*p* < 0.001) and intermediate (*p* < 0.001) CR-P groups and steeper 2-year decline in the low (*p* = 0.044), intermediate (borderline significance: *p* = 0.059), and high (*p* = 0.042) CR-P groups. Second, we have a latent E-CR factor compared with other studies that have used only single variables (i.e., education) as a proxy for CR. This approach examines the common variances across two IQ variables (WAIS vocabulary, NART-R) and education to eliminate any measurement errors associated with testing procedures and single variables. In addition, our latent factor in Mplus by default accounts for missing data with maximum likelihood estimation to generate factor scores. Third, to the best of our knowledge, this is the first study to distinguish between E-CR versus L-CR, and importantly our results show that such distinction accounts for additive effects of CR that is accumulated throughout the lifespan. This has not always been emphasized in studies on CR. Fourth, compared with previous CR studies, we stratified by sex to examine differences in CR moderation of ventricular volume in men and women. Fifth, we examined a commonly used global measure of cognitive performance and decline. The DRS includes several subscales of cognitive domains, including executive function, memory, and attention, and is widely used to test clinical performance in dementia [44].

## Conclusions

We examined independent and synergistic contributions of E-CR (education, IQ) and L-CR (occupation) on the association between neurodegeneration (ventricular volume) and dementia severity as stratified by sex. Larger baseline ventricular size was associated with poorer DRS baseline performance and steeper 2-year DRS decline in a heterogeneous neurodegenerative population (i.e., AD, VCI, combined). This association was moderated by E-CR (education and IQ), L-CR (occupation, which may represent a form of maintenance), and the additive effects (CR-P) of E-CR and L-CR. All moderations were differentially represented by sex. Specifically, E-CR significantly moderated the association between ventricular size and DRS performance in men and L-CR in women. Men with high CR-P were protected from the detrimental effects of poorer baseline and steeper 2-year DRS decline with larger baseline ventricular size. However, women with high CR-P showed steeper 2-year DRS decline with larger baseline ventricular size, and this rate of decline was magnified for women with low CR-P. Future studies should consider replicating this important contribution in other samples. In dementia, complex interactions between CR (in early life and across the life course) and neuroimaging biomarkers (i.e., ventricular atrophy) on cognitive impairment may be selective and differentially represented in men and women. Future research should focus on similar innovative methodological approaches to identify potential complex and dynamic CR mechanisms that may lead to personalized interventions across neurodegenerative diseases.

## Additional file

Additional file 1: **Table S1.** Frequencies of participants for Hollingshead Index of Social Position (occupation scale) by sex. **Table S2.** Baseline participant characteristics by clinical diagnoses and sex. **Table S3.** Baseline participant characteristics by clinical diagnoses and *Apolipoprotein E* (*APOE*) status (ɛ4−/ɛ4+). (DOCX 32 kb)

## Abbreviations

β: Regression estimate
${x}_M^2$: Chi-square test of model fit
*df*_*M*_: Degrees of freedom for model fit
AD: Alzheimer’s disease
*APOE*: *Apolipoprotein E*
CFI: Comparative fit index
CR: Cognitive reserve
CR-P: E-CR + L-CR
DLB: Dementia with Lewy bodies
DRS: Dementia Rating Scale
E-CR: Early-life cognitive reserve
FTD: Frontotemporal dementia
*i*: Intercept
ICV: Intracranial volume
L-CR: Later-life cognitive reserve
MCI: Mild cognitive impairment
NART-R: North American Adult Reading Test–Revised
RMSEA: Root mean square error of approximation
*s*: Slope
SDS: Sunnybrook Dementia Study
SRMR: Standardized root mean square residual
*T*: Time point
VCI: Vascular cognitive impairment
WAIS: Wechsler Adult Intelligence Scale
WMH: White matter hyperintensities
