# Supplementary material for: Clinical dementia severity associated with ventricular size is differentially moderated by cognitive reserve in men and women
Source: Alzheimers Res Ther. 2018 Sep 5;10:89. doi: 10.1186/s13195-018-0419-2 (PMC6123907; doi:10.1186/s13195-018-0419-2)
Supplement: Supplementary file 1 — Table S1. Frequencies of participants for Hollingshead Index of Social Position (occupation scale) by sex. Table S2. Baseline participant characteristics by clinical diagnoses and sex. Table S3. Baseline participant characteristics by clinical diagnoses and Apolipoprotein E (APOE) status (ɛ4−/ɛ4+). (DOCX 32 kb) [file 13195_2018_419_MOESM1_ESM.docx]

**Additional file 1**

Table S1. Frequencies of participants for Hollingshead Index of Social Position (occupation scale) by sex.

| **Occupation Scale** | **Men** | **Women** | **Total** |
| --- | --- | --- | --- |
| Higher executives of large concerns, proprietors, and major professionals (1) | 60 | 14 | 74 |
| Business managers, proprietors of medium-sized businesses, and lesser professionals (2) | 101 | 98 | 199 |
| Administrative personnel, owners of small businesses, and minor professionals (3) | 66 | 62 | 128 |
| Clerical and sales workers, technicians, and owners of little businesses (4) | 54 | 130 | 184 |
| Skilled manual employees (or light manual labor) (5) | 11 | 24 | 35 |
| Machine operators and semi-skilled employees (or heavier manual labor) (6) | 45 | 5 | 50 |
| Unskilled employees (or hard physical work) (7) | 8 | 28 | 36 |

Table S2. Baseline participant characteristics by clinical diagnoses and sex.

| **Characteristics** |  |  |  |  |  |  |
| --- | --- | --- | --- | --- | --- | --- |
| **Men** | **AD** | **MCI** | **VCI** | **FTD** | **LBD** | **Total** |
| *n* | 206 | 30 | 32 | 61 | 20 | 349 |
| Age | 71.6 (9.2) | 71.7 (10.4) | 72.1 (7.4) | 66.2 (9.3) | 72.4 (8.3) | 70.7 (9.3) |
| MMSE | 22.9 (5.2) | 27.2 (2.1) | 24.7 (3.8) | 23.6 (5.9) | 26.0 (2.0) | 23.8 (5.0) |
| Ventricular size (cm^3^)^a^ | 46.4 (22.4) | 35.3 (17.6) | 47.7 (26.0) | 39.4 (17.9) | 45.3 (21.5) | 44.3 (21.9) |
| WMH (cm^3^)^a^ | 7.0 (9.6) | 3.0 (4.0) | 9.7 (10.4) | 3.8 (5.8) | 7.8 (14.0) | 6.4 (9.2) |
| TIV (cm^3^) | 1302.1 (120.8) | 1278.7 (110.2) | 1262.9 (124.9) | 1269.4 (113.6) | 1336.1 (86.9) | 1292.7 (118.3) |
| Dementia Rating Scale | 116.4 (16.4) | 131.4 (9.8) | 122.4 (13.9) | 118.0 (17.2) | 124.9 (8.7) | 119.2 (16.1) |
| NART-R | 106.7 (10.7) | 109.0 (9.9) | 106.1 (11.4) | 101.6 (11.0) | 110.2 (10.7) | 106.3 (10.9) |
| WAIS vocabulary | 42.8 (15.0) | 53.8 (8.9) | 39.4 (12.9) | 39.4 (18.6) | 46.0 (15.3) | 43.0 (15.0) |
| Education | 14.2 (4.2) | 13.8 (3.5) | 13.8 (4.3) | 14.8 (4.0) | 14.2 (3.3) | 14.2 (4.1) |
| *APOE (*ɛ4-/ɛ4+) | 73/93 | 7/9 | 21/2 | 27/16 | 8/6 | 136/126 |
| **Women** | **AD** | **MCI** | **VCI** | **FTD** | **LBD** | **Total** |
| *n* | 233 | 47 | 20 | 64 | 10 | 374 |
| Age | 71.9 (9.4) | 69.5 (9.6) | 69.6 (9.3) | 67.7 (9.0) | 74.8 (10.0) | 70.8 (9.5) |
| MMSE | 23.1 (4.4) | 27.9 (1.7) | 27.4 (3.4) | 22.7 (6.1) | 19.8 (5.2) | 23.8 (4.8) |
| Ventricular size (cm^3^)^a^ | 40.8 (16.9) | 28.9 (16.7) | 42.2 (22.1) | 45.4 (23.7) | 37.4 (16.4) | 40.1 (18.9) |
| WMH (cm^3^)^a^ | 8.46 (12.2) | 3.48 (5.3) | 18.7 (24.3) | 5.4 (7.6) | 6.1 (4.8) | 7.8 (12.1) |
| TIV (cm^3^) | 1135.4 (105.3) | 1146.4 (99.5) | 1164.7 (92.0) | 1143.5 (93.5) | 1136.1 (81.3) | 1139.7 (101.2) |
| Dementia Rating Scale | 119.0 (13.1) | 134.8 (5.9) | 132.6 (11.0) | 113.5 (21.0) | 106.3 (16.5) | 120.5 (15.7) |
| NART-R | 108.1 (9.1) | 110.8 (8.6) | 110.9 (8.2) | 102.9 (11.2) | 109.0 (10.1) | 107.9 (9.6) |
| WAIS vocabulary | 42.7 (13.2) | 50.0 (10.1) | 46.9 (10.9) | 31.9 (17.9) | 41.0 (5.7) | 42.5 (14.1) |
| Education | 13.5 (3.4) | 14.1 (3.3) | 13.5 (3.2) | 13.5 (3.6) | 13.3 (3.7) | 13.6 (3.4) |
| *APOE (*ɛ4-/ɛ4+) | 69/104 | 16/11 | 10/3 | 36/8 | 3/3 | 134/129 |

^a^corrected for total intracranial volume.

*Abbreviations.* *AD* Alzheimer’s disease, *MCI* Cognitive Impairment, *VCI* Vascular Cognitive Impairment, *FTD* Frontotemporal Dementia, *LBD* Lewy Body Disease, *MMSE* Mini-Mental State Exam, *WMH* White matter hyperintensities, *TIV* Total Intracranial Volume, *NART-R* National Adult Reading Test-Revised, *WAIS* Wechsler Adult Intelligence Scale, *APOE* *Apolipoprotein E*.

Table S3. Baseline participant characteristics by clinical diagnoses and *Apolipoprotein E* (*APOE*) status (ɛ4-/ɛ4+).

| **Characteristics** |  |  |  |  |  |  |
| --- | --- | --- | --- | --- | --- | --- |
| ***APOE* ɛ4-** | **AD** | **MCI** | **VCI** | **FTD** | **LBD** | **Total** |
| *n* | 142 | 23 | 31 | 63 | 11 | 270 |
| Age | 72.1 (10.7) | 69.6 (9.7) | 70.1 (9.2) | 66.9 (9.8) | 69.9 (8.4) | 70.4 (10.3) |
| Sex (f/m) | 69/73 | 16/7 | 10/21 | 36/27 | 3/8 | 134/136 |
| MMSE | 23.2 (4.6) | 27.8 (2.0) | 25.7 (4.1) | 22.8 (5.8) | 24.1 (3.7) | 23.9 (4.9) |
| Ventricular size (cm^3^)^a^ | 45.0 (22.4) | 30.1 (15.8) | 42.5 (25.6) | 43.1 (21.2) | 34.4 (12.9) | 42.6 (22.0) |
| WMH (cm^3^)^a^ | 8.3 (11.8) | 3.5 (6.2) | 9.7 (11.3) | 4.3 (6.3) | 4.9 (4.9) | 7.0 (10.3) |
| TIV (cm^3^) | 1218.1 (138.6) | 1180.3 (112.7) | 1235.6 (140.2) | 1205.3 (116.1) | 1267.2 (80.0) | 1215.9 (130.2) |
| Dementia Rating Scale | 117.7 (15.3) | 134.4 (7.9) | 124.3 (14.4) | 115.5 (18.7) | 119.4 (10.4) | 119.5 (16.2) |
| NART-R | 106.8 (10.8) | 110.7 (9.7) | 109.9 (9.5) | 101.1 (11.5) | 105.4 (7.1) | 106.3 (11.0) |
| WAIS vocabulary | 41.8 (13.8) | 46.0 (13.7) | 40.9 (12.2) | 32.0 (16.9) | 45.0 (7.2) | 40.3 (14.4) |
| Education | 14.0 (3.6) | 14.7 (3.4) | 14.7 (3.5) | 13.7 (4.0) | 13.6 (3.0) | 14.1 (3.7) |
| ***APOE* ɛ4+** | **AD** | **MCI** | **VCI** | **FTD** | **LBD** | **Total** |
| *n* | 197 | 20 | 5 | 24 | 9 | 255 |
| Age | 71.0 (8.6) | 69.9 (10.1) | 73.3 (7.2) | 66.2 (7.9) | 73.7 (9.8) | 70.6 (8.7) |
| Sex (f/m) | 104/93 | 11/9 | 3/2 | 8/16 | 3/6 | 129/126 |
| MMSE | 23.3 (4.6) | 27.1 (1.6) | 24.0 (5.2) | 25.6 (4.2) | 24.2 (5.3) | 23.8 (4.6) |
| Ventricular size (cm^3^)^a^ | 42.4 (19.3) | 31.4 (13.8) | 66.4 (20.7) | 38.7 (19.0) | 37.9 (18.1) | 41.5 (19.3) |
| WMH (cm^3^)^a^ | 7.2 (9.5) | 3.0 (4.4) | 14.2 (12.5) | 4.9 (7.6) | 5.4 (4.0) | 6.8 (9.0) |
| TIV (cm^3^) | 1221.0 (142.0) | 1215.4 (105.8) | 1187.0 (71.4) | 1229.4 (122.5) | 1273.2 (176.9) | 1222.5 (137.6) |
| Dementia Rating Scale | 118.9 (13.9) | 133.6 (6.2) | 119.2 (19.0) | 119.7 (15.5) | 119.6 (14.7) | 120.1 (14.2) |
| NART-R | 108.0 (9.7) | 108.3 (10.3) | 103.9 (12.4) | 102.9 (11.6) | 113.7 (11.1) | 107.7 (10.1) |
| WAIS vocabulary | 44.5 (14.3) | 56.8 (4.8) | 38.0 (16.5) | 36.6 (23.0) | 47.0 (2.8) | 44.4 (14.9) |
| Education | 13.8 (3.6) | 14.4 (2.7) | 11.6 (2.6) | 15.4 (3.2) | 14.0 (4.3) | 13.9 (3.5) |

^a^corrected for total intracranial volume.

*Abbreviations.* *AD* Alzheimer’s disease, *MCI* Cognitive Impairment, *VCI* Vascular Cognitive Impairment, *FTD* Frontotemporal Dementia, *LBD* Lewy Body Disease, *MMSE* Mini-Mental State Exam, *WMH* White matter hyperintensities, *TIV* Total Intracranial Volume, *NART-R* National Adult Reading Test-Revised, *WAIS vocabulary* Wechsler Adult Intelligence Scale.
